# Dissecting the mechanisms of squirrel monkey (*Saimiri boliviensis*) social learning

**DOI:** 10.7717/peerj.13

**Published:** 2013-02-12

**Authors:** LM Hopper, AN Holmes, LE Williams, SF Brosnan

**Affiliations:** 1 Lester E. Fisher Center for the Study and Conservation of Apes, Lincoln Park Zoo, Chicago, IL, USA; 2 Language Research Center, Georgia State University, Atlanta, GA, USA; 3 Michale E. Keeling Center for Comparative Medicine and Research, UT MD Anderson Cancer Center, Bastrop, TX, USA; 4 Department of Psychology & Neuroscience Institute, Georgia State University, Atlanta, GA, USA

**Keywords:** Squirrel monkey, *Saimiri*, Emulation, Social facilitation, Social learning, Ghost display

## Abstract

Although the social learning abilities of monkeys have been well documented, this research has only focused on a few species. Furthermore, of those that also incorporated dissections of social learning mechanisms, the majority studied either capuchins (*Cebus apella*) or marmosets (*Callithrix jacchus*). To gain a broader understanding of how monkeys gain new skills, we tested squirrel monkeys (*Saimiri boliviensis*) which have never been studied in tests of social learning mechanisms. To determine whether S. boliviensis can socially learn, we ran “open diffusion” tests with monkeys housed in two social groups (*N* = 23). Over the course of 10 20-min sessions, the monkeys in each group observed a trained group member retrieving a mealworm from a bidirectional task (the “Slide-box”). Two thirds (67%) of these monkeys both learned how to operate the Slide-box and they also moved the door significantly more times in the direction modeled by the trained demonstrator than the alternative direction. To tease apart the underlying social learning mechanisms we ran a series of three control conditions with 35 squirrel monkeys that had no previous experience with the Slide-box. The first replicated the experimental open diffusion sessions but without the inclusion of a trained model, the second was a no-information control with dyads of monkeys, and the third was a ‘ghost’ display shown to individual monkeys. The first two controls tested for the importance of social support (mere presence effect) and the ghost display showed the affordances of the task to the monkeys. The monkeys showed a certain level of success in the group control (54% of subjects solved the task on one or more occasions) and paired controls (28% were successful) but none were successful in the ghost control. We propose that the squirrel monkeys’ learning, observed in the experimental open diffusion tests, can be best described by a combination of social learning mechanisms in concert; in this case, those mechanisms are most likely object movement reenactment and social facilitation. We discuss the interplay of these mechanisms and how they related to learning shown by other primate species.

## Introduction

Broadly speaking, when encountering an unknown environment, an individual can learn about it either through individual, trial-and-error learning, or by observing knowledgeable conspecifics and learning from their behavior, or the outcomes of their behavior. The latter – social learning – can take a number of forms, termed social learning mechanisms (Whiten et al., 2004; Whiten et al., 2009; Bates & Byrne, 2010; Hopper, 2010). At the simplest level, an individual’s attention may be drawn to a particular object or locale by the presence of another individual (“stimulus/local enhancement”) or the presence of a conspecific could encourage exploration by the naïve individual (“mere presence effect”, Caldwell & Whiten, 2003). Alternatively, an individual may learn about the physical properties of their environment from the outcomes of another’s actions (Byrne, 1998). In this way, the naïve individual can learn how to solve the problem themselves by “emulating”; achieving the same end-state but via novel means (Wood, 1989). Conversely, an animal may “imitate”, and copy the actions of a conspecific (Whiten et al., 2009), which has been proposed to allow for the most faithful transmission (Tennie, Call & Tomasello, 2009, but see Caldwell et al., 2012 for a review of high fidelity transmission via emulation).

Despite our increasing knowledge about the social learning mechanisms employed by a broad spectrum of species, including apes (e.g., Call, Carpenter & Tomasello, 2005), dogs (e.g., *Canis familaris*, Miller, Rayburn-Reeves & Zentall , 2009), pigs (e.g., *Sus scrofa,* Oostindjer et al., 2011), rats (e.g., *Rattus rattus*, Zohar & Terkel, 1991), birds (e.g., *Columba livia,* Klein & Zentall, 2003), and insects (e.g., *Bombus impatiens*, Leadbeater & Chittka, 2007), there are surprisingly few data available for monkeys. Although the social learning abilities of monkeys have been well documented, both in captivity (e.g., Dindo, Thierry & Whiten, 2008; Dindo, Whiten & de Waal, 2009a; Kendal, Coe & Laland , 2005; Price & Caldwell, 2007) and in the wild (e.g., Perry, 2009), as noted by Fragaszy & Visalberghi (2004), this research has only focused on a few species. Furthermore, the majority of studies that have incorporated dissections of social learning mechanisms were run with either capuchins (*Cebus apella* e.g., Dindo, Whiten & de Waal, 2009b) or marmosets (*Callithrix jacchus*, e.g., Bugnyar & Huber, 1997; Voelkl & Huber, 2000, but see also Subiaul et al., 2004 for a study with *Macaca mulatta*). Little is therefore known about the social learning mechanisms of other monkey species.

In contrast to apes, previous research has suggested that monkeys are most likely to rely on “simpler” forms of social learning mechanisms (e.g., stimulus enhancement or social facilitation) rather than imitation (Fragaszy & Visalberghi, 2004; Hecht et al., 2012; but see Price & Caldwell, 2007 for a discussion of possible imitation by *Colobus guereza kikuyuebsus*). As research with monkeys is still in its infancy, further studies are required to elucidate the social learning mechanisms and strategies that typify monkeys. To address this, and to complement previous studies with capuchins and marmosets, we tested an understudied New World primate, the squirrel monkey (*Saimiri boliviensis*). As no study has attempted to tease apart the social learning mechanisms employed by squirrel monkeys (*Saimiri* spp.), this study would both provide a much-needed detailed inspection of this less well studied species while also enabling a broader understanding of the general capacity for social learning by New World monkeys (the group that includes the better-studied capuchins and marmosets, discussed above).

We selected *S. boliviensis* as our study species because they are a highly gregarious species that live in large multi-male, multi-female groups in the wild (Mitchell, 1994), which provides the perfect environment for social learning. Without direct empirical evidence, however, we cannot assume that they can socially learn just because they are highly sociable (Reader & Lefebvre, 2001). For example, although *S. boliviensis* do live in large social groups, individuals typically only associate with members of their own age and sex class (Mendoza, Lyons & Saltzman, 1991) which may inhibit the spread of socially-learned behaviors. Promisingly, observations of wild monkeys suggest that they can socially learn and, as has been proposed for chimpanzees, such learning may be mediated by the complexity of the skill they are trying to acquire (Hopper et al., 2010). Specifically, squirrel monkeys appear to ignore social information pertaining to simple skills (e.g., selecting which fruit to eat, Boinski & Fragaszy, 1989) but do look to others when faced with more complex actions (e.g., the capture of live insect prey, Boinski & Timm, 1985, but note that this could also be because prey are more engaging for the monkeys to look at than are fruit).

Perhaps then, social learning is not essential for enabling squirrel monkeys to learn *which* foods to eat, but does become important when learning *how* to eat (i.e. when the food item is more cryptic and/or requires greater processing, as for insect prey). Therefore, rather than just testing whether squirrel monkey food preferences are socially induced (Visalberghi & Addessi, 2000), we were interested to ascertain whether they used social information to learn how to process a food item. To test this experimentally, we made the food item (in this case, a mealworm) more difficult to obtain by encasing it in a puzzle box or “artificial fruit” (c.f. Whiten et al., 1996). With this apparatus, we wished to answer two questions: can *S. boliviensis* socially learn and, if so, what mechanism typifies their learning?

First, to determine whether *S. boliviensis* can socially learn, we ran “open diffusion” tests (c.f. Whiten, Horner & de Waal, 2005) with 23 monkeys housed in two social groups (we acknowledge that although we tested over 20 monkeys, the number of groups tested was small, *N* = 2, but this is comparable to previous open diffusion tests with other primate species e.g., Bonnie et al., 2007; Hopper et al., 2007; Whiten, Horner & de Waal, 2005). For these, a member of each social group was trained to perform a specific method for removing the defense from the artificial fruit. The task used was the bidirectional “Slide-box” (Hopper et al., 2008; Hopper, Lambeth & Schapiro, 2012, Figure 1) which has a door that can be moved to either the left or right to reveal a food reward. The remaining monkeys in the group were then allowed to observe the trained monkey use the Slide-box. In this way, we could monitor whether the monkeys were able to learn this action from observing a trained model and, if so, whether this introduced behavior spread throughout the group. Such open diffusion paradigms have been used successfully in a number of studies with both apes (e.g., Bonnie et al., 2007; Whiten, Horner & de Waal, 2005; Whiten et al., 2007) and monkeys (e.g., Dindo, Whiten & de Waal, 2009a; Kendal, Coe & Laland , 2005; Price & Caldwell, 2007) but no truly unconstrained open diffusion study has ever been run with squirrel monkeys before (we note that Messer EJ, Claidiere N, Hoppitt W, & Whiten A (unpublished data) recently reported a comparable method with *S. sciureus*, but as they restricted the number of monkeys that could observe the model at any one time, their study more closely mirrors the “replacement method” e.g., Menzel, Davenport & Rogers, 1972).

Second, if social learning was observed, we wished to tease apart the underlying social learning mechanisms. In these experimental groups, after observing the trained monkey, the observers may learn that they can move the Slide-box door and they may also copy the direction in which they saw it move. If this occurred, there are a number of social learning mechanisms that could explain this transfer and matching. To determine which was at play we ran a series of three control conditions with 35 squirrel monkeys that had no previous experience with the Slide-box in order to tease apart the relative importance of social facilitation, emulation and imitation. Following the “two-method, three-group” design employed by Whiten, Horner & de Waal (2005), the first two control conditions (Group Control and Pair Control) were designed to provide the monkeys with social support but no information about how to solve the task. This was to test whether potential successful learning was facilitated by mere presence effects or stimulus enhancement.

If the monkeys matched the direction that the door was moved, rather than more generally learning that “the door could move”, their learning could be described as emulative or imitative. Previous, studies with nonhuman primates have employed a number of different techniques to distinguish imitation and emulation, both through the incorporation of specific experimental protocols and through *post hoc* analysis. One technique, specifically developed to distinguish emulative from imitative learning, is the “ghost” display (Hopper, 2010). In this, the movements, or affordances, of a task are shown to a naïve observer without showing a model acting upon the task (e.g., Huang & Charman, 2005; Hopper et al., 2007; Tennie, Call & Tomasello, 2010). In this manner, a ghost display can reveal whether the observer is able to reach the same goal, or end-state, without seeing the actions required to reach it (Tomasello, 1999; Whiten et al., 2009). The ghost display was first used in the 1970s (Groesbeck & Duerfeldt, 1971), although the term was not coined until 2002 (Fawcett, Skinner & Goldsmith, 2002), and has been used in tests of social learning mechanisms with a number of species including primates (Hopper et al., 2008); birds (Klein & Zentall, 2003); dogs (Miller, Rayburn-Reeves & Zentall , 2009); rats (Heyes et al., 1994), and humans (Thompson & Russell, 2004).

Specifically, in our “Ghost Control”, the door on the Slide-box was slid back and forth discretely with monofilament fishing line (c.f. Hopper et al., 2008). This revealed to the observing monkey (i) how the door could move, (ii) a particular direction of travel and, (iii) that a food reward could be obtained if the door of Slide-box was moved away from the central position. If the monkeys copied the direction of door travel in the experimental group conditions and this Ghost Control then the most parsimonious explanation for the matching in both would be emulation (object movement reenactment, Whiten et al., 2004). If the monkeys only showed faithful matching after seeing a conspecific operate the Slide-box, however, then we would conclude that the presence of the live model was vital for learning to occur. Such action copying could be considered indicative of imitation (Hopper et al., 2007, but see Tennie, Call & Tomasello, 2009 for an alternative explanation). Following the majority of previous social learning studies which have incorporated ghost controls, both with primates and non-primate species (e.g., Heyes et al., 1994; Hopper et al., 2007; Miller, Rayburn-Reeves & Zentall , 2009, see Hopper, 2010 for a review), we tested the monkeys individually in order to remove potential learning via social facilitation or from the mere presence of having a social partner (Hopper et al., 2008).

Given the limited information from observations of wild *S. boliviensis*, we had no firm predictions as to whether *S. boliviensis* would evidence social learning nor did we have directional predictions about which social learning mechanisms they would employ if capable of social learning.

## Materials and Methods

### Ethics statement

The subjects for this behavioral study were 58 socially-housed squirrel monkeys at the Michale E Keeling Center for Comparative Medicine and Research, UT MD Anderson Cancer Center, Bastrop, TX, USA (“UT MD Anderson” hereafter). This study was approved by IACUC (ACUF ID #: 03-12-04281) and UT MD Anderson is fully accredited by AAALAC-I. For all experimental conditions, throughout testing periods, in addition to the food rewards that the monkeys could obtain from the test apparatus, they had *ad libitum* access to both food and water and were never food or water deprived at any time.

### Subjects and housing

#### Experimental group open diffusion

Twenty-three squirrel monkeys (*Saimiri boliviensis*), socially-housed at UT MD Anderson, were the subjects for this study. The squirrel monkeys were housed in two groups, designated the “push-left” and “push-right” groups. The push-right group housed 10 monkeys (9 females, 1 male, average age = 4.1 years, range 1–15 years) and the push-left group housed 13 monkeys (11 females, 2 males, average age = 2.9 years, range 1–10 years).

We note that the two experimental groups for this study were comprised predominantly of females with a few infant males. The social make-up of these groups was designed to reflect wild groups of *S. boliviensis* in which males emigrate from their natal group (Boinski, 1999) and there is little to no interaction between females and males during the nonbreeding season (Williams & Abee, 1988). Furthermore, both in captivity and the wild, female *S. boliviensis* are known to direct aggression toward adult males (Boinski, 1999) and so reducing the number of males within a group can help to alleviate this. This is especially important for a study such as this in which access over a single resource (in this case the test apparatus) may have exacerbated aggression (something that we wished to avoid).

Each group lived in large, highly-enriched, cages (1.2 × 1.8 × 4.3 m) with many climbing structures to encourage three dimensional use of the cage. Enrichment devices, which were changed every two weeks, were hung throughout and were designed to encourage manipulation and exploration. In addition to the food that the monkeys could obtain for the test apparatus, the monkeys were fed one meal of fresh produce and two meals of New World Primate Diet (Lab Diet, PMI Nutrition International) per day.

### Group control

This group of 13 monkeys comprised 11 females and two males (average age = 2.9 years, range 1–9 years) and was housed in identical conditions as described for the Experimental Groups (tested in the open diffusion condition).

### Pair control

Fourteen pair-housed monkeys (8 males and 6 females, average age = 4.3 years, range 1–11 years) were selected to act as pair controls to test for the influence of social facilitation. These pairs were housed in highly enriched caging (158 × 76 × 64 cm) and they followed the same feeding schedule as the group-housed monkeys. Each pair was comprised of monkeys that were familiar with each other.

### Ghost control

Eight female monkeys (average age = 6.4 years, range 5–8 years) were presented with ghost displays (see Procedure below for more details) to test for the importance of object movement reenactment. For their test, these monkeys were transferred from their large home cage to a smaller cage for their individual tests and returned to their group directly after the completion of the test. Similar to the criteria set for model monkeys to be trained for the Experimental Group Open Diffusion tests (see below), only those monkeys that were comfortable with being isolated (i.e. were calm and would accept food once isolated) were used as subjects for this test condition.

### Apparatus

The task used for all conditions was a modified Slide-box, a bidirectional task used previously in tests designed to tease apart the social learning mechanisms of chimpanzees and children (Hopper et al., 2008; Hopper, Lambeth & Schapiro, 2012, Figure 1). To ensure that the Slide-box was suitable for use with squirrel monkeys we made a smaller version (the front panel was 40 × 30 cm) with a door (5 cm^2^) that was constructed from a lighter plastic so that it would be easier for the squirrel monkeys to manipulate. The front panel of the Slide-box was transparent plastic. This feature enabled the experimenters to see and code which monkeys (i) operated the task or (ii) observed cagemates move the door. The exception to the clear front was the central door, which was made of opaque red plastic so that when in the central “start” position, it obscured the platform directly behind it that held the food reward (a mealworm). The door was set on runners so it could move equally well to the right or the left, and either action revealed the mealworm behind the door. This meant that all actions were reinforced equally. Furthermore, the design of this task meant that stimulus enhancement alone could not cue the monkeys which direction to push the door, because the door was in the same starting position for both actions.

**Figure 1:**
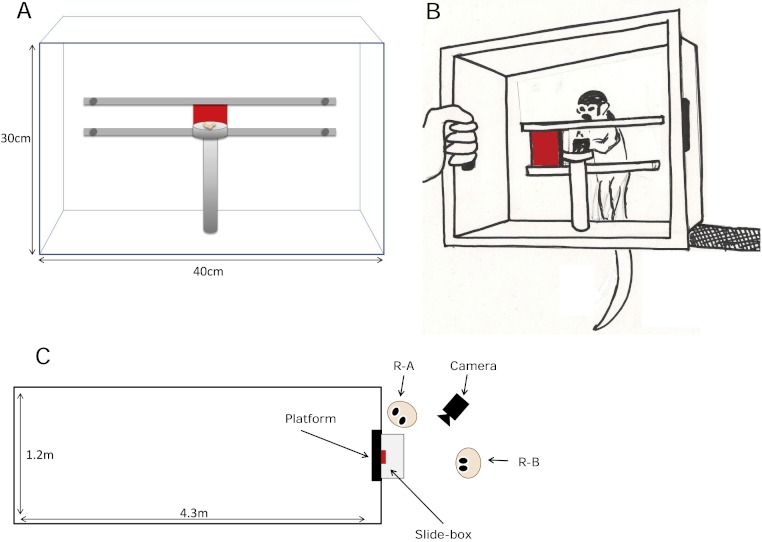
The Slide-box and experimental set-up. (A) A diagram of the Slide-box, from the researcher’s perspective. The Slide-box is shown in the “start” position with the door in the center of the runners. With the door in this position it obscures the mealworm, placed on the food platform, from the monkey in the cage behind. (B) A drawing of a squirrel monkey retrieving a meal worm from the Slide-box having pushed the door to her right. For all tests an experimenter held the Slide-box against the mesh of the monkeys’ cage. The Slide-box was held at 80 cm above the ground so that it was a level with a platform within the monkeys’ cage. This was done so that the monkeys could stand on the platform (see Fig. 1C) while they either used the Slide-box or observed another monkey use the Slide-box. (C) An overview of the experimental set-up. One of the two researchers (R-A) held the Slide-box up against the monkeys’ cage while the second researcher (R-B) stood directly behind the Slide-box. R-A not only held the Slide-box, but they were also responsible for baiting the Slide-box with a meal worm and re-setting the door of the Slide-box between trials. R-A coded which monkey moved the door of the Slide-box and in which direction they moved the door. R-B coded the identity of all monkeys classed as “observers” for each trial. A video camera was placed so that it had direct sight of the Slide-box and the monkeys behind.

### Procedure

#### Experimental group open diffusion

A monkey from each group was selected to act as the model. In the push-right group this monkey was trained to push the door of the Slide-box to the right and in the push-left group the model was trained to push the door to the left. These model monkeys were selected according to the following criteria: they had to be (i) dominant enough to use the task without being usurped and (ii) comfortable being isolated for training.

To test the first criterion we presented the monkeys with a clumped food patch on their feeding platform for 10 min (Boccia, Laudenslager & Reite, 1988). During this period we noted which animals readily approached the platform and took food and which of those were able to maintain their position and not be displaced by group members. Animals were considered to be dominant if they could maintain access to the food platform regardless of which other monkeys approached. To test the second criterion, we moved the monkey that appeared the most dominant to a cage that adjoined to their home cage. As we did not want their group mates to see the movement of the Slide-box door during the model training sessions, this “training cage” had opaque sides. In this cage, the monkey being trained was physically separated from their group but still had auditory contact. The monkey that was placed in the training cage was then observed by two experimenters. If the monkey seemed calm and would readily take food from the experimenters, they continued on with their training schedule. If, however, the separated monkey would not take food and/or appeared agitated they were immediately returned to their home cage. Each potential model was given a maximum of two periods in the training cage. If they did not appear calm in either session we commenced this procedure with the monkey that had been rated as the next most dominant. For the push-right group, a three-year-old female was first selected as the model but as she did not appear calm in the training cage we ultimately selected a one-year-old male as the model. The first monkey we selected for the push-left group, a two-year-old female, passed both criteria and was that group’s model. We note that the first female sampled, but ultimately rejected, for the role of model for the push-right group never received any experience with the Slide-box. When initially isolated to explore whether she would be a good model, she was never calm enough to take food from the experimenters, and so we did not commence any training with her. Therefore this female had no individual experience with the Slide-box which could have influenced her responses when exposed as an observer in the test sessions. This is critical to verify that all observers were naïve about the task and only received information from the trained model.

To train each of the models we used a shaping procedure with positive reinforcement (Pryor, 2009; Gillis, Janes & Kaufman, 2012). We first presented the monkey with the Slide-box with the door slid all the way to the edge of the runners appropriate to the door movement that we were training them on. For example, for the monkey being trained to push-left, they were first shown the Slide-box with the door pushed all the way to the left. The monkey was first rewarded for removing the food reward from the Slide-box without having to touch the door. Incrementally, we then moved the door so that it covered more and more of the food-reward hole. In this way, the monkey would have to reach past the door to gain the food reward. This continued until the monkeys were comfortable with touching the door and, eventually, with moving it out of the way to gain the reward.

The food rewards used during this training were mealworms, pieces of mini marshmallow and pieces of grape (different to food rewards used during test sessions). Each training session lasted no more than 10 min and, at all other times, both monkeys were housed with their group mates in their home cage. These monkeys each required approximately ten sessions to become familiarized with the training cage and to learn the required behavior. Models were considered “trained” when he/she was able to push the door of the Slide-box in their designated direction ten times in succession without attempting to move the door in the opposite direction.

Both of the experimental groups were given ten 20-min open diffusion sessions. The experimental groups were never tested more than once per day. The Slide-box was presented to the monkeys such that the door was always in the start position in the center of the runners, obscuring the food reward. For the first test session, to ensure that naïve group members observed the seeded technique, and following previous such studies with chimpanzees (e.g., Whiten, Horner & de Waal, 2005; Hopper et al., 2007), only the trained model was allowed to use the task for the first ten responses. During this period, if another monkey attempted to use the slide box, the experimenter moved it out of reach. After this controlled observation period, the whole group of monkeys was always allowed free access to interact with the task throughout each of the 20-min sessions. Ultimately, each group received ten 20-min sessions run over a period of two weeks for each group. During these test periods, if a monkey moved the door of the Slide-box they were allowed to get the mealworm on the food tray, following which the experimenter returned the door of the Slide-box to the central position, out of sight of the group to avoid cuing any of the monkeys, and the task was then re-presented to monkeys.

### Group control

The methods for the Control Group were the same as those for the two experimental open diffusion groups except for the fact that there was no trained model monkey. The food reward used throughout was mealworms.

### Pair control

Pair-housed monkeys were presented with the Slide-box with no form of demonstration and were given a ten-minute period of free-access with the task (this time was set following the period of time we have used in other social learning controls with primates, e.g., Hopper, Lambeth & Schapiro, 2012). These monkeys were allowed to move the door as many times as they could during this ten minute period. All responses were recorded in real-time by the experimenter, including the identity of the monkey which moved the door.

### Ghost control

For the ghost display four of the monkeys were shown push-left and the other four were shown push-right. For both, the experimenter tied a length of monofilament fishing-line to one side of the door so that it could be moved discretely in one direction (*sensu* Hopper et al., 2008). To emulate the scrounging that occurred during the group open diffusion sessions (in both the experimental and control groups), for every fifth door-movement the monkey was allowed access to the reward from the food tray. In this condition monkeys were individually shown 20 ghost displays and were then given a ten-minute free-access period identical to that for the Pair Control, with the exception that the monkeys were singly-housed for this test.

### Coding and analysis

The responses of the monkeys in all conditions were coded in real-time but each test was also filmed to enable post hoc coding and analysis if required. Two experimenters (LMH and ANH) were present and involved during the running of each test. For every condition the experimenters coded (i) if a monkey moved the door, (ii) the identity of that monkey and (iii) in which direction they moved the door. In the group sessions we also coded which animals observed their cage mates move the door of the Slide-box, including the identities of all individuals involved in using the box or observing another monkey use it.

During the open diffusion tests (the two experimental groups and the Group Control), the two experimenters took turns acting in one of two roles: “model coder” or “observer coder”. The model coder was responsible for baiting the apparatus with a mealworm and holding the Slide-box up against the caging so that the front face was flush with the cage mesh. This person also recorded which monkey manipulated the Slide-box door and in which direction they moved it. The second experimenter, the observer coder, recorded every monkey that was within the 40 cm^2^ area created by the front face of the Slide-box door. This area was easily coded as any monkey which had at least their head within the perimeter of the apparatus, and was oriented towards the Slide-box, was coded as an observer.

Each monkey wore a color-coded ID tag, the color of which indicated the age of the monkey. Also on these tags was written the monkey’s unique ID number. These were all clearly visible and enabled the experimenters to quickly and clearly identify individual monkeys. To ensure accuracy, prior to running any of the experimental or control tests, the two experimenters ran a series of pilot coding sessions with groups of monkeys that were not tested for this study. For these sessions, both experimenters practiced acting in the roles of model coder and observer coder to ensure that they could accurately and reliably identify the squirrel monkeys. No testing occurred until both coders were in complete agreement in their score sheets (i.e. both coders were coding the identities of monkeys with 100% agreement). The movement of the Slide-box door was extremely clear and easy to code and so there was never disagreement between the two experimenters with regard to this. Furthermore, once the monkeys moved the door in a particular direction they never returned it to its central, starting, position and so when the experimenter removed the Slide-box to reset the door (out of sight of the monkeys) she could further verify the direction it had been moved if needed.

Due to the small sample sizes, to compare the level of success (i.e. whether the monkeys moved the door) across conditions we used Fisher’s Exact Tests. When comparing the level of matching between the two experimental group conditions we employed the Univariate General Linear Model (SPSS), as not all monkeys made the same number of responses. All tests were two-tailed. To account for potential family-wise errors arising from multiple cross-condition comparisons, we applied a bonferroni correction. When comparing the two experimental groups with the group control we set our α = 0.03 (α = 0.05/3 conditions).

## Results

### Experimental group open diffusion

**Figure 2:**
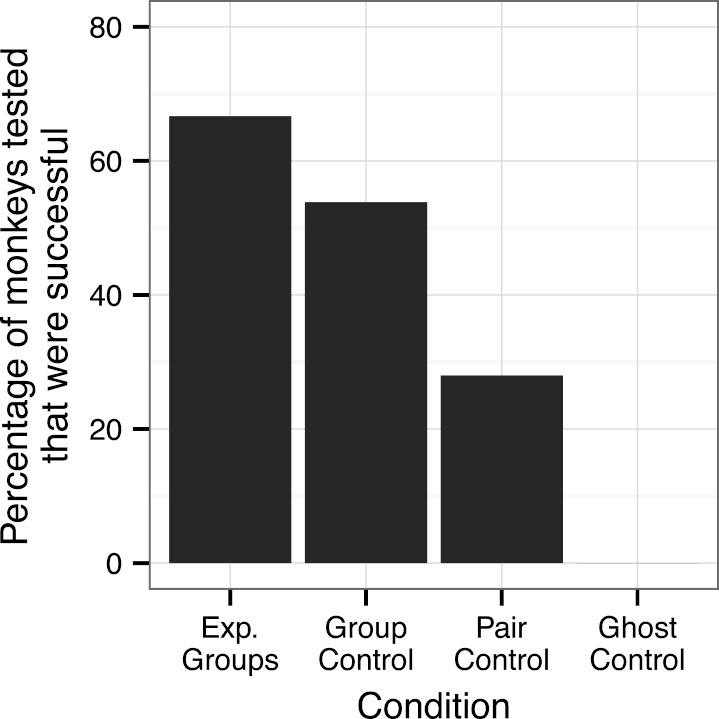
The percentage of monkeys that had one or more successful response when presented with the Slide-box. Where “Exp. Groups” represents the two Experimental Groups combined and includes the two monkeys in the experimental push-right group were successful only when the dominant female was removed from their group.

**Figure 3:**
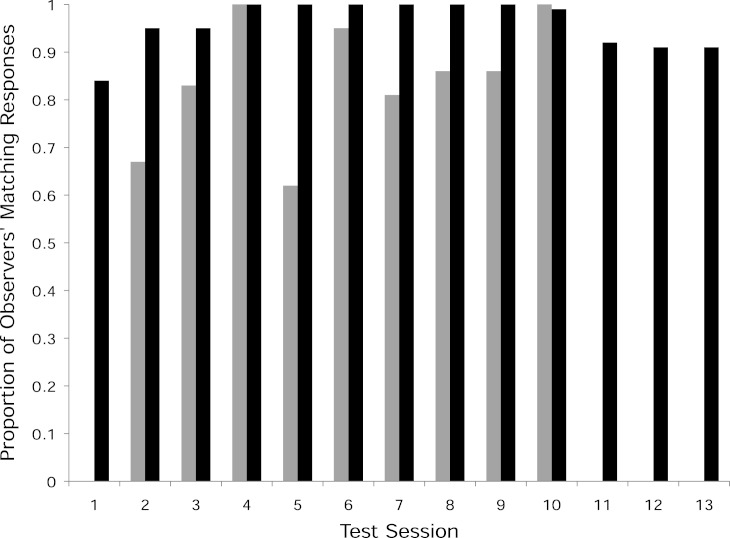
Proportion of matching responses. The proportion of the responses in each test session that matched the seeded door-movement direction (gray bars = push-left group, black bars = push-right group). Also shown are the three additional open diffusion sessions (11–13) that the push-right group received with the dominant female removed from the group. Note, no observer monkey in the push-left group responded in the first test session as the model monkey in that group dominated the task. The lack of matching responses shown, therefore, indicates a lack of response per se, rather than a failure to copy the model during this session.

In the two experimental groups, 12 of the 21 observer monkeys successfully moved the door on the Slide-box at least once to gain a mealworm (5/9 in the push-right group and 7/12 in the push-left group, Fig. 2). There was no difference between these two groups in terms of the number of successful subjects (Fisher’s Exact Test, *P* > 0.05). Of the monkeys who solved the task, individuals were more likely to push in the direction of their demonstration than the other direction; in the push-right group, a greater proportion of the observer monkeys’ responses were “push-right” compared to the responses of monkeys in the push-left group (Univariate GLM by GROUP, Type III Sum of Squares: *F* = 8.22, *d* *f* = 1, *P* = 0.0015, Fig. 3).

In the push-right group, after learning how to operate the Slide-box in the first test session, one 9-year-old female monopolized the task and her responses accounted for 61% of the responses made by the observer monkeys across the 10 sessions. It is well documented that it is adult females who control *S. boliviensis* group cohesion (Williams & Abee, 1988; Mendoza, Lyons & Saltzman, 1991), and so it is not surprising to note that the second most prolific monkey in this group was this dominant female’s two-year-old daughter (who accounted for 37% of the observers’ responses). This did not occur in the push-left group, in which the monkey to make the most responses aside from the model only accounted for 23% of the group’s responses.

Therefore, once the 10 sessions had been completed, and to test whether other monkeys in the push-right group could operate the task when it was not being monopolized, we ran a further three sessions (total time = 1 h) with this dominant female removed from the group. Across these three additional sessions, two monkeys used the Slide-box for the first time, thus in this group seven of the nine observer monkeys were ultimately successful at using the Slide-box. Including these two new monkeys, there was still no difference in the number of successful monkeys in each group (Fisher’s Exact test, 7/9 *versus*  7/12, *P* > 0.05).

Considering the level of matching in the push-right group, including these two new monkeys that responded in the absence of the dominant female, the group made a higher proportion of push-right responses than did those seven in the push-left group (Univariate GLM by GROUP, *F* = 6.779, *d* *f* = 1, *P* = 0.021). As shown in Fig. 3, the matching to the seeded method shown by the group was maintained in these three additional test sessions, which confirms that the strength of the matching in the initial ten sessions reflects the group’s response as a whole.

Considering the individual responses of the monkeys in the experimental groups, seven of the observers that responded showed significant matching of the model across all their responses (Table 1). Although this only represents half of the 14 successful monkeys, we note that four more monkeys also showed high proportion of matching across all their responses (3 = 100% and 1 = 88%), but due to the small number of responses they made, it was not significant (Table 1). If we were to include these monkeys then 79% (11/14) of the monkeys showed individual matching to their model’s seeded method, following the overall pattern shown by the two groups when considered at a group-level.

**Table 1:** Individual responses of monkeys in the experimental groups. The individual responses of the monkeys from the two experimental groups when tested in the open diffusion tests. For each group, the monkeys are listed in order of acquisition. “% of Group” is the percentage of the group’s total responses that each individual contributed. “% Match” is the percentage of each monkey’s total responses that matched the model’s method. Whether this matching was significant is shown in the “Significant Matching?” column. Binomial tests were used to compute whether the monkeys’ responses significantly matched the model. Note, some monkeys made so few responses (<2) that such analysis could not be run.

Condition	Monkey	Number Match	Number not Match	% of Group	% Match	Significant Matching?
Push-right	Model	68	1	8.0	99.0	<0.001
	A	313	5	36.6	98.4	<0.001
	B	408	4	47.5	99.0	<0.001
	C	28	6	3.9	82.4	0.002
	D	20	4	2.8	83.3	0.002
	E	7	1	1.0	87.5	0.070
	F	2	0	0.2	100.0	0.500
	G	0	1	0.1	0.0	–
Push-left	Model	286	29	55.0	90.8	<0.001
	A	0	1	0.2	0.0	–
	B	107	22	22.5	83.0	<0.001
	C	4	0	0.7	100.0	0.125
	D	0	3	0.5	0.0	0.250
	E	35	6	7.2	85.4	<0.001
	F	74	5	13.8	93.7	<0.001
	G	1	0	0.2	100.0	–

### Group control

In the Group Control, across all ten sessions, 7 of the 13 monkeys successfully used the box at least once (Fig. 2). There was no difference in the success of these monkeys as compared to those in the two experimental groups: Push-right *versus* Group Control (5/9 *versus*  7/13, Fisher’s Exact Test: *P* > 0.05) and Push-left *versus* Group Control (7/12 *versus*  7/13, *P* > 0.05), including the extension sessions with the push-right group (7/9 *versus*  7/13, *P* > 0.05).

Monkeys in the push-right group made a greater proportion of push-right responses than the monkeys in the Group control (*F* = 7.830, *d* *f* = 1, *P* = 0.019). In contrast, there was no significant difference between the proportion of push-left responses made by those monkeys in the push-left group compared to the Group Control (*F* = 2.005, *d* *f* = 1, *P* = 0.182). When the responses of the two models in the experimental groups are included, there was still no difference between the proportion of push-left responses made by monkeys in the push-left group and the Group Control (*F* = 2.772, *d* *f* = 1, *P* = 0.120). This is likely explained by the propensity of monkeys in the Group Control to slide the door to the left (of the total 610 responses made during the ten sessions by monkeys in the Group Control 376, or 62%, were push-left).

### Pair control

Of those 14 monkeys tested in the Pair Controls, four were successful at moving the door of the Slide-box in their ten-minute response period (Fig. 2). Of these four, three made a successful response without any prior information about the Slide-box. The fourth monkey was a test partner of one of three successful monkeys, so it cannot be determined whether this monkey discovered the solution via social or asocial means. Therefore, we can only say with certainty that three monkeys did not rely on observational learning to solve the task (21%). We note that the mere presence of their partner may have enhanced their success, but they certainly did not gain knowledge about the mechanisms of the task from their partner.

In the ten minutes available to them, four monkeys in the Pair Control successfully operated the Slide-box while in the first ten minutes that the monkeys in the Group Control had access to the Slide-box, six were successful. Considering just this ten-minute period, there was no difference in the number of successful monkeys in either condition (4/14 *versus*  6/13, Fisher’s Exact Test, *P* > 0.05).

### Ghost control

None of the eight monkeys tested in the Ghost Control were successful in their ten-minute response period (Fig. 2); significantly less than those in the Group Control (0/8 *versus* 6/13, *P* = 0.045) and the two experimental groups combined (0/8 *versus*  14/21, *P* = 0.002). The monkeys in the ghost control, however, were no less successful than those in the Pair Control (0/8 *versus*  4/14, *P* > 0.05).

Comparable Ghost Control tests with the Slide-box have previously been run with chimpanzees and children (see Hopper et al., 2008 for full details). In these previous tests, both the chimpanzees and children, like the squirrel monkeys, were tested individually and had no prior exposure to the task. It is notable that all subjects tested (eight chimpanzees and eight children) made one or more successful responses in this test, whether or not they matched the direction of the door movement. In this condition, therefore, both chimpanzees and children showed greater success than did the squirrel monkeys in the present study (0/8 *versus*  8/8, Fisher’s Exact Test, *P* < 0.001 for both).

## Discussion

When squirrel monkeys observed a trained member of their social group move the door on the Slide-box to retrieve a mealworm, two thirds (67%) were then also able to successfully acquire a meal worm from the Slide-box. Furthermore, these successful individuals showed a significant level of matching; those in the push-right group moved the door more often in the same direction as that used by their group’s model than the alternative direction. This success, and matching, could potentially indicate that the monkeys were learning this new skill via social learning, something confirmed by the three control conditions. Furthermore, we can rule out potential genetic and environmental confounds which may explain differences in responses of the two experimental groups. Greater scrutiny has recently been given to the behavioral traditions of wild chimpanzees, with some data showing environmental (Humle & Matsuzawa, 2002, but see Schöning et al., 2008) or genetic (Langergraber et al., 2011, but see Lycett, Collard & McGrew, 2007) influences on their “cultural” behaviors. This called into doubt the whether these traditions are reliant on social learning (see also Tennie, Call & Tomasello, 2009). With our captive study of squirrel monkeys, however, we were able to rule out environmental (all monkeys were housed at the same facility, in identical cages, fed the same food and provided with the same enrichment) and genetic (we reviewed the kinship coefficients both between and within the groups and determined that they were not significantly different) differences. Any differences in methods for operating the Slide-box, therefore, are most likely to be socially learnt (Hopper et al., 2007) or at least maintained by social learning (Tennie, Call & Tomasello, 2009).

When faced with a bidirectional task, such as the Slide-box, the monkeys could have solved the task in several ways. First, they could have merely copied the direction that they observed the door move and then recreated that movement themselves (i.e. object movement reenactment) or second, they may have been influenced by the physical actions of their group’s model (Whiten et al., 2004). Through the inclusion of the Ghost Control, which highlighted to the monkeys how, and in what direction, the door could move, we can tentatively rule out the former; none of the monkeys were able to replicate the door movement in the absence of a conspecific demonstrator. We note that this failure may be due to the reduced social support available to these monkeys in the Ghost Control (Tennie, Call & Tomasello, 2010). Indeed, studies with chimpanzees have shown that chimpanzees are more likely to learn in tests of emulation when they are tested with a conspecific companion (e.g., Hopper et al., 2008) compared to when tested alone (Hopper et al., 2007, although differences in task complexity may also explain the differences across these two studies, Caldwell et al., 2012). Accordingly, at this stage, we only draw tentative conclusions from the responses of the monkeys in the Ghost Control and propose that future tests should be run in which monkeys are tested with the social support of a conspecific partner, as in the Pair Control (see also Caldwell et al., 2012; Hopper et al., 2008; Klein & Zentall, 2003 for examples such “enhanced” ghost controls), to determine the interplay between social facilitation and object movement reenactment.

Perhaps then, mere presence effect or social facilitation – increased investigation of one’s environment facilitated by the presence of a conspecific – is the most likely social learning mechanism to explain the success of the monkeys, rather than emulation. Not only did the monkeys in the two experimental groups show elevated success compared to those in the Ghost Control, but just over half (54%) the monkeys in the Group Control, and just over a quarter (28%) of the monkeys in the Pair Control, were successful on one or more occasion. Social facilitation not only provides the most parsimonious explanation for the monkeys’ success (Hopper & Whiten, 2012), but it has previously been shown to account for learning success by other New World monkeys (capuchins: Dindo, Whiten & de Waal, 2009b; marmosets: Caldwell & Whiten, 2003). Furthermore, the mere presence of conspecifics has been shown to reduce squirrel monkeys’ adrenocortical response to stress (Stanton, Patterson & Levine, 1985). Perhaps those monkeys tested with conspecifics experienced less stress when presented with the Slide-box, were less neophobic, and so were more likely to explore (and solve) the task.

As some monkeys were able to solve the Slide-box without the need for a demonstration by a model conspecific, it could be argued that this particular task falls within their “Zone of Latent Solutions” (Tennie, Call & Tomasello, 2009). Simply put, as some, although not all, members of a group could learn the Slide-box, it is within the species’ innate abilities. Tennie, Call & Tomasello (2009) proposed that innovation could arise from invention by “particularly gifted individuals” (p. 2406), from whom group members could socially learn, and a behavioral tradition could propagate. Following the criteria of the Zone of Latent Solutions, this was true for the squirrel monkeys we tested with the Slide-box (i.e. as shown by monkeys in the Group Control) but we also reserve caution when extrapolating the success of a few individuals to describe an entire group or species (Thornton & Lukas, 2012). Ultimately, we argue, the Slide-box represents a suitably complex task that could not be solved out-right by trial-and-error learning by the majority of the monkeys tested (Hopper et al., 2010): success was limited to a few specific individuals who could discover it by themselves (c.f. Tennie, Call & Tomasello, 2009) or those that were able to observe group members already proficient in using it.

Furthermore, neither individual learning, nor social facilitation, explains the matching of the model’s door-push direction in the two experimental groups (as reported for other monkey species, e.g., Price & Caldwell, 2007). Despite the arbitrary nature of the task, and the potential ease of its solution, when the monkeys were in their home group they nonetheless selected the particular method also used by their group mates, indicating a tendency to base their responses on those of their group mates’ (see also Bonnie et al., 2007; Hopper et al., 2011). We propose that this matching was most likely facilitated by object movement reenactment (Whiten et al., 2004) but that the presence of conspecifics (social facilitation) encouraged the squirrel monkeys’ interactions with the task which resulted in this matching (Dindo, Whiten & de Waal, 2009b).

The matching of the seeded method, by monkeys in the experimental groups, is also emphasized by a bias observed among monkeys in the control groups to push the door to the left (62% of responses in the Group Control and 86% of responses in the Pair Control were “push-left”). Conversely, of the responses of the observers in the push-right group, 661 of their 671 responses (99%) were push-right. Thus the push-right monkeys were potentially changing their behavior as compared to a push-left bias, rather than just chance. This push-left bias may be explained by population-level right-handedness. If a monkey had a manual preference for their right hand, it would be easiest for them to slide the door of the Slide-box to the left. Unfortunately, in the present study, we were unable to record which hand each monkey used as they manipulated the Slide-box (this was because the monkeys moved so quickly and we could only code the identity of the monkeys, not the hand they used, and also from the video footage it was impossible to discern, with certainty, which hand a monkey used to move the Slide-box door). Research into squirrel monkey handedness is sparse and the evidence mixed (King & Landau, 1993; Roney & King, 1993; Aruguete, Ely & King, 2005). However, without the data to show which hand the monkeys used we cannot be certain. We merely offer this as a “working hypothesis” for why the monkeys showed this bias and we encourage future research to investigate the laterality in squirrel monkeys.

Ultimately, we propose that the squirrel monkeys’ learning can be best described by a combination of social learning mechanisms in concert; in this case, object movement reenactment (for matching) and social facilitation (for solving the task). Previous research with nonhuman primates has often aimed to distinguish specific social learning mechanisms from others, or even to define species by a particular learning mechanism (e.g., that chimpanzees are “emulators”; Call, Carpenter & Tomasello, 2005). It seems to us that social learning mechanisms need not be mutually-exclusive and indeed that they often occur simultaneously (see Hopper, 2010 for a review). Furthermore, it may be that, rather than particular species predominantly relying on one particular mechanism, each has a suite of methods through which they can learn, and can employ differentially depending on the environment (both physical and social, Hopper et al., 2010). Thus, even for this simple task, there may have been multiple mechanisms at play, one allowing the monkeys to solve the task, and another that facilitated matching. We encourage future research to test more groups of monkeys and also to run additional dissections of their social learning mechanisms.
